# Quality of Urban Green Space for Older Adults to Promote Physical Activity: A Systematic Review

**DOI:** 10.3390/ijerph23080970

**Published:** 2026-07-27

**Authors:** Nargis Sultana, Kathryn L. Braun

**Affiliations:** 1 University of Hawai’i at Mānoa Library, University of Hawai’i at Mānoa, Honolulu, HI 96822, USA; nargis@hawaii.edu; 2 Thompson School of Social Work & Public Health, University of Hawai’i at Mānoa, 1960 East-West Road, Biomed D-209, Honolulu, HI 96822, USA

**Keywords:** aging, older adults, urban green spaces, physical activity, built environment, park accessibility

## Abstract

**Highlights:**

**Public health relevance—How does this work relate to a public health issue?**

- Promoting physical activity in older adults helps support their independence by maintaining functional ability and overall health.
- The review considers urban green space (UGS), a key element of urban design, as an intervention to promote physical activity in older adults.

**Public health significance—Why is this work of significance to public health?**

- This review examines how different dimensions of UGS (availability, accessibility, and attractiveness) influence physical activity in older adults.
- The review identifies key sources of heterogeneity, including variations in definitions of older adults, measurement approaches, and socioeconomic and geographic contexts of studies, which limit comparability across findings.

**Public health implications—What are the key implications or messages for practitioners, policymakers and/or researchers?**

- More research is needed, especially qualitative and elder-engaged studies, to design UGS to support physical activity in this age group.
- Future research and policy should prioritize age-friendly design, standardized measures, and inclusive planning approaches to support active aging.

**Abstract:**

Regular physical activity (PA) is essential for healthy aging. For older adults residing in urban areas, access to urban green space (UGS) can help increase PA levels. This systematic review examined the association between availability, accessibility, and attractiveness of the UGS on PA among older adults. PubMed, CINAHL, and Web of Science were searched for articles published 2016–2024 that used quantitative methods to explore associations between the qualities of UGS and measures of PA in this demographic. Findings from nine studies from Europe, China, Hong Kong, and Australia were synthesized by availability, accessibility, and attractiveness domains. For availability, the presence and size of UGS were positively associated with older adults’ PA in several studies, whereas UGS proximity was inconsistently associated with PA. For accessibility, infrastructure-related features such as trail length were positively associated with PA, but findings for park features and safety were mixed. Results were also inconsistent for attractiveness; some land-cover and amenity features showed positive associations, while others showed no clear relationship with PA. In addition to variability in associations, there was great heterogeneity across studies in age inclusion criteria (e.g., 45+ vs. 65+), participant inclusion criteria, setting, operationalization of UGS quality, PA measures, and study rigor, reducing the ability to draw conclusions about the association between UGS qualities and PA. Although findings can begin to inform urban planners, public health professionals, and policymakers in designing UGS to promote PA in older adults, the field would benefit from more consistent definitions and measures of UGS qualities and PA.

## 1. Introduction

Regular physical activity (PA) can prevent or delay many health problems that become increasingly common with aging, including heart disease, cancer, hypertension, diabetes, arthritis, and dementia [1]. For older adults, PA improves strength and flexibility, strengthens motor skills, and reduces the risk of falling and fracturing bones, all of which help maintain the ability to live independently [2]. Regular PA also improves cognitive function and lowers the risk of vascular dementia in older adults [3].

Adults over 65 are advised to engage in 150 min of moderate-intensity activity weekly [1]. Unfortunately, research has established that people become less active and more prone to sedentary behavior as they age [4,5,6,7]. Globally, 27.5% of adults did not meet PA recommendations in 2022, posing a significant mortality risk [5].

Around the world, the number of adults aged 65+ living in urban settings increased from 160 million to 355 million between 1990 and 2015, and the number will rise to 1.5 billion by 2050 [8]. The built environment around older people significantly impacts their levels of PA and sedentary behavior [9,10]. Improving access to built environments that promote an active lifestyle can enhance the overall well-being of the aging population.

The World Health Organization (WHO) recommends using urban green space (UGS) as an intervention to enhance urban dwellers’ health and well-being [11]. Numerous studies have found that UGS can improve health and well-being by providing a setting to promote PA, mental and psychological relaxation, and social connections, as well as being a source of oxygen for breathing and purifying air pollutants [11,12,13,14]. A US-based study also found that the presence of neighborhood green space lowers healthcare costs [15].

While UGS can be used to create an inclusive environment for all ages to be physically active, research findings have also pinpointed disparities in UGS accessibility and emphasized the need to assess the quality aspects of UGS for health, specifically for vulnerable groups like older adults [15,16,17,18]. The quality of UGS refers to the attributes of UGS that impact the pattern of use and interaction with UGS, including characteristics (e.g., size or proximity), features (e.g., facilities or amenities), and the fitness of purpose (e.g., maintenance or condition) [19].

Three published reviews [9,20,21] explored the relationship between older adults’ health and UGS [9], open spaces and parks [20], and physical environments [21]. The review by Xu et al. [9] explored UGS characteristics or qualities and the subjective well-being of older adults. Review findings suggested that key features of UGS that significantly enhance the well-being of older adults included accessibility, the park type and size, and biodiversity, which promote PA and social interactions. Additionally, the quality, safety, and environmental features, such as shade and pleasant microclimates, of these spaces can further support or hinder use and mental health outcomes.

Cauwenberg et al. [21] reviewed articles on the overall physical environment and concluded that knowledge about the relationship between the physical environment and PA in older adults was limited. Few studies have reported significant relationships, but this could have been due to methodological issues within the studies. They recommended that future research use standardized, reliable, and validated measures for the physical environment, as well as for PA.

Levy-Storms et al. [20] reviewed articles on the impact of open space and parks on PA. They concluded that parks with walkable paths, shaded areas, seating, restrooms, and proper lighting are attractive to older adults and promote PA. However, key barriers to park use included safety concerns, accessibility issues, and a lack of age-friendly park designs. The review pointed out the limitations of the included studies, including reliance on correlational study designs, inconsistent measures of PA, and a lack of research on diverse populations and longitudinal impacts. They also found that some cities (e.g., Shanghai, Taipei, and London) are redesigning parks to include age-friendly exercise equipment and dedicated senior areas. In contrast, U.S. cities have fewer recreational spaces designed specifically for older adults.

The three reviews share similar limitations, including reliance on quantitative research methods that may not capture personal experiences and perceptions of UGS or the physical environment. Reliance on survey data and correlational analyses limited the depth of the data and the understanding of the complex interactions affecting the health and well-being of older adults. Furthermore, the studies included in these reviews relied on self-reported data for health outcomes and lacked longitudinal data collection, reducing the generalizability of the findings. Among the three reviews, only Levy-Storms et al. [20] reviewed the quality/characteristics of parks and open spaces that influenced PA among older adults.

### Conceptual Framework

Authors who have explored the causal relationship between UGS and various health-related outcomes have considered three main qualities or characteristics of UGS—availability, accessibility, and attractiveness [22,23,24]. These three aspects of UGS were adopted from a causal model of the impacts of UGS on health and well-being presented by the WHO in 2017 [25].

**Figure 1:**
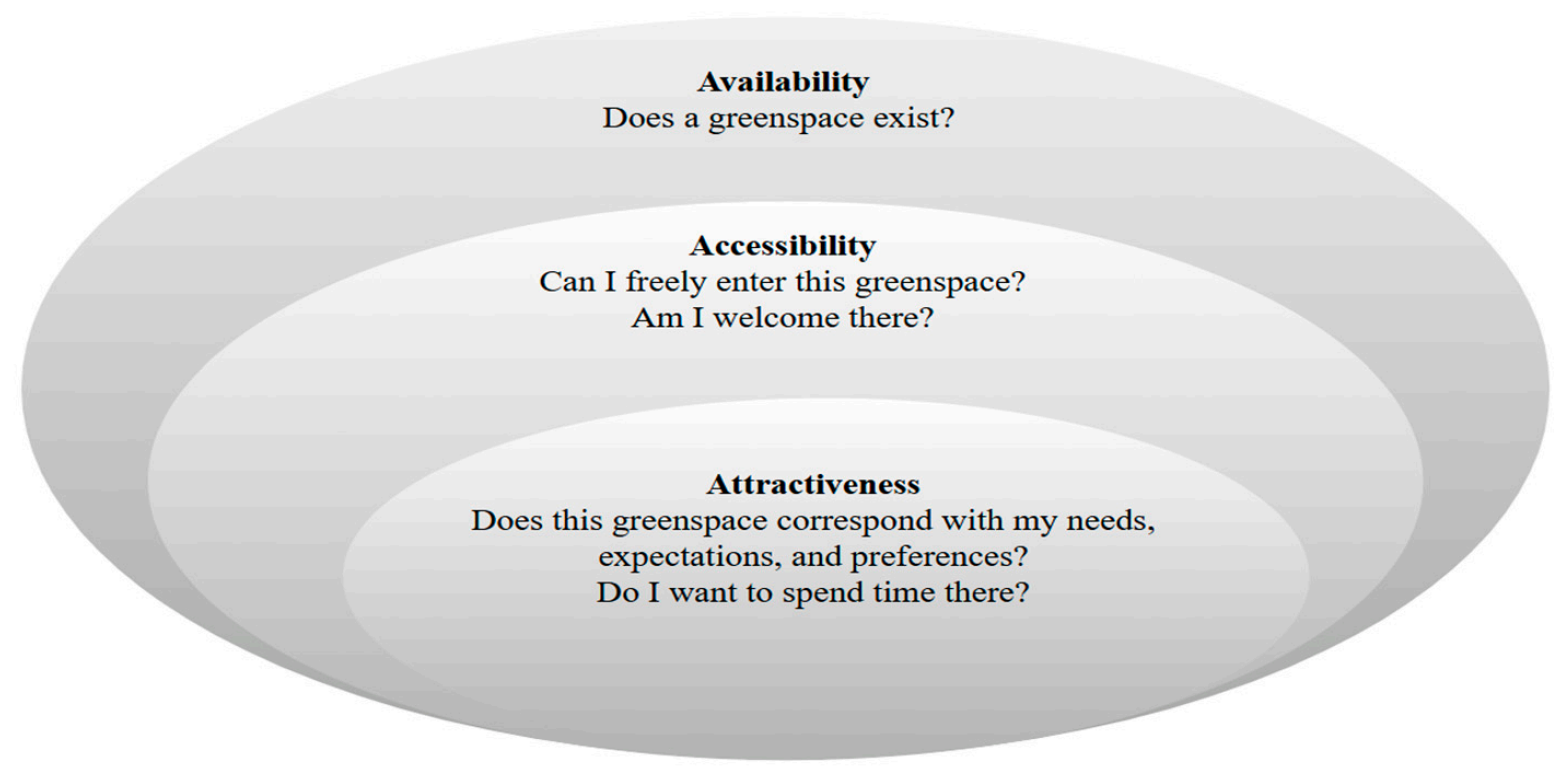
Three dimensions of UGS provision for barrier-free access [22].

Biernacka and Kronenberg [22,23] organized these three aspects hierarchically, as shown in Figure 1. In this framework, availability refers to the presence of green spaces in an area, the distribution of parks, and proximity to green spaces within walking or driving distance. The author argued that availability is a prerequisite of accessibility, which refers to both physical and psychological aspects of accessibility. Physical accessibility relates to the ease of entry, and psychological accessibility relates to the psychological barriers, such as feeling unsafe or unwelcome, that may discourage use of UGS. Thus, a UGS is considered accessible if a person can reach and enter freely without any restrictions and if it has the necessary infrastructure, such as barrier-free entry, walking paths, bike lanes, car parking spaces, guiding signage, adaptations for disabled users, public transport connections, and road safety. Therefore, an assessment of UGS quality that enhances accessibility should encompass not only physical or spatial attributes but also consider and reflect on the perceived characteristics of the population.

Attractiveness is the third quality. Attractiveness is related to the preferences and expectations of the UGS users. A UGS is considered attractive when people want to use it and spend time there, particularly when the environment aligns with their personal requirements, expectations, and preferences. This includes features related to the general maintenance and cleanliness of the area, the presence of amenities (e.g., benches, playgrounds, restrooms, water fountains, and public toilets), as well as the primary surface materials (e.g., pavement vs. ground or grass). This framework aligns with the WHO’s recommendations for UGS [11]. This review used this conceptual model to explore how UGS qualities support PA among urban older adults.

Building on this conceptual framework, the goal of this review was to examine the published literature to explore how the availability, accessibility, and attractiveness of UGS promote PA among older adults. In this paper, the term UGS is used as an umbrella term, encompassing parks, playgrounds, green spaces, greenways, sports fields, recreation areas, and public grounds.

## 2. Materials and Methods

The Preferred Reporting Items for Systematic Reviews and Meta-Analysis (PRISMA) statement [26] was followed to ensure transparent, comprehensive, and reproducible search, screening, selection, review, and synthesis processes. The review was not registered, as PROSPERO does not allow students to register reviews conducted as academic assignments.

### 2.1. Search Strategy and Search Terms

A systematic search was performed by one of the authors (N.S.) in three electronic databases, PubMed, CINAHL, and Web of Science, to gather evidence of effectiveness from the latest studies. The search terms were constructed using the PICOS (participants, interventions, comparisons, outcomes, and studies) framework (Table 1).

For “population,” keywords such as aged, older adults, elderly, and seniors were used. Although older adults are frequently defined as being aged 60 and older or 65 and older [1], in this review, the definition of older adults was accepted as defined by the studies. For “intervention,” the databases were searched using the following terms: UGS, parks, playground, green space, greenspace, greenway, sports field, recreation area, public ground, public parks, and outdoor. This review included studies that considered any quality or characteristic of UGS, such as proximity, density, size, pattern, features (facilities, amenities), conditions (safety, maintenance), or design improvements. For “outcomes,” the following keywords were used: exercise, physical activity, physical health, walking, cycling, biking, bicycling, active play, leisure, recreating, and sports.

Boolean logic, “AND” and “OR” was used to perform searches. The exact search strings and Boolean logic used to perform the search are available in the Supplementary Materials (S1).

### 2.2. Inclusion and Exclusion Criteria

To be included in the review, the studies must have included measures of PA, which could include subjective and/or objective measures of overall PA, walking- and cycling-specific measures, or recreation measures in older adults. The “study design” was limited to quantitative studies to compare the statistical direction of the relationships between quality elements of UGS and older adults’ PA. Articles were only included if they were published in English, peer-reviewed, and available in full text. Additionally, since the Levy-Storms et al. [20] review included articles published through 2015, this review was limited to the articles published from 2016 to 2024.

Studies that did not examine any availability, accessibility, or attractiveness quality of UGS in the analysis were excluded. Studies that evaluated activities in national parks were excluded because national parks are usually in regional settings and often have an entry fee, whereas the goal of this review was to evaluate the impact of no-cost UGS at a local level. Systematic reviews, literature reviews, scoping reviews, study protocols, and conference abstracts were excluded because this review included only primary studies with original data. Reviews were excluded to avoid duplication, while protocols and conference abstracts were excluded because they do not provide complete study results or enough methodological detail for analysis. Also excluded were articles not published in English and those not published between 2016 and 2024. Studies that used a qualitative study design were also excluded to allow a comparison of the strength of the outcome among the selected studies.

### 2.3. Study Screening and Selection

The keywords were used to identify articles. The retrieved articles were then exported to Zotero (version 6), a reference manager software, and duplicate articles were removed. The titles and abstracts of the remaining articles were screened in Rayyan (version 1.4.3), a systematic review screening software. Those that did not fulfill the eligibility criteria were removed through title and abstract screening. Articles that met the eligibility criteria were further assessed by reading the full text, and the inclusion and exclusion criteria were again applied to remove articles from the final selection.

### 2.4. Data Extraction and Analysis

MS Excel was used to organize the extracted relevant information for evidence. The information extracted from the articles included the place/country where the study was conducted, study population age group and sex distribution, study design, sample size, outcome measured, UGS type, UGS quality, tools used to measure UGS quality, PA outcome measures, and tools used to measure PA outcomes. Extracted information was reviewed by both authors. Information from retrieved articles was organized and analyzed based on the hierarchy dimension framework recommended by Biernacka and Kronenberg [22] (Figure 1).

### 2.5. Study Quality Assessment Tool

Appraisal checklists by the Joanna Briggs Institute (JBI) were used to evaluate the methodological rigor of each study [27]. The JBI offers specific checklists to address the quality of various study designs. In this systematic review, the appropriate JBI checklist was used, and each item from the JBI checklist was classified as met, not met, or not clear. The percentage of total items met was calculated to compare the studies that used different study designs.

## 3. Results

**Figure 2:**
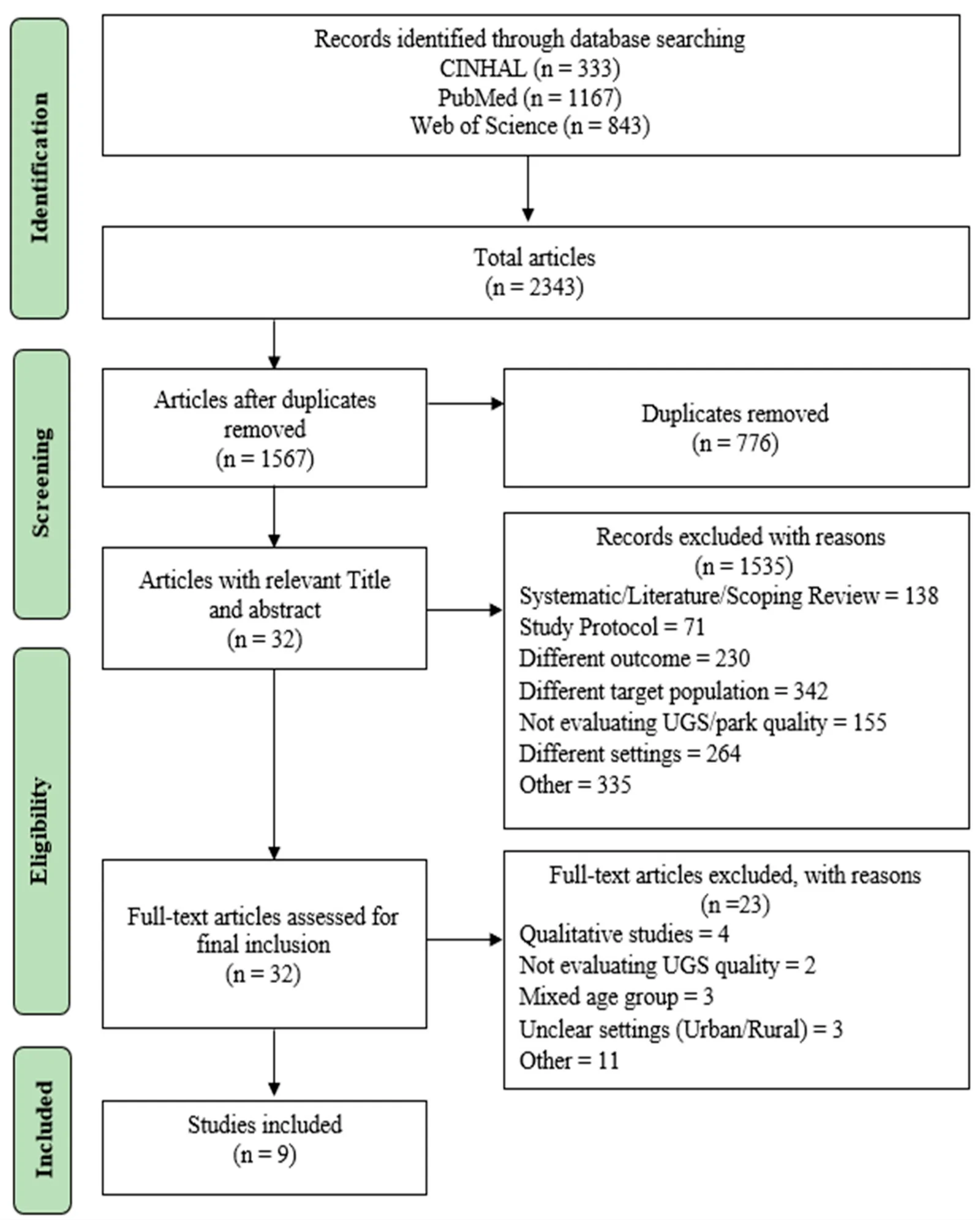
PRISMA flow diagram of selection of studies for review.

The initial search yielded 2343 articles. After removing duplicates, 1567 unique articles were included for the title and abstract review, from which 32 articles were identified for full-text screening. Using inclusion and exclusion criteria, another 23 articles were excluded for not meeting the eligibility criteria. Nine articles were included in the final review [28,29,30,31,32,33,34,35,36] (Figure 2).

### 3.1. General Description of the Selected Studies

Six of the nine articles were published in environment- and health-related journals (e.g., *International Journal of Environmental Research and Public Health*). The remaining three articles were retrieved from journals dealing with the urban green environment (e.g., *Urban Forestry & Urban Greening*, *International Journal of Urban Policy and Planning*). The nine articles were published between 2018 and 2022.

Table 2 details the general characteristics of each study. The featured studies were conducted in five different countries. One study was conducted in cities in Australia [28]; four studies were conducted in China [30,31,33,36]; three were conducted in cities in the European region—one in Belgium [29], one in the United Kingdom [35] and one in Spain [32]—and one was a comparison study between Hong Kong and Leipzig, Germany [34].

Studies differed in their inclusion criteria regarding participants’ ages. Four studies included participants aged 60 years and older [30,31,33,34]; three studies limited participation to those 65 years and older [29,35,36]; one considered people aged 45 years and above as older adults [28]; and one did not mention any age group but just used the term “elderly” to describe its participants from senior centers [32]. Eight of the nine studies used a cross-sectional study design to examine the relationship between UGS quality and PA outcomes, and one used a longitudinal research design [29]. Study sample sizes varied from 122 to 18,094. The percentage of female participants was between 42 and 57 percent.

The participants of the studies were selected from various places within urban areas. Park users were considered as the participants in three studies [30,34,36]. One study included older adult residents with Type 2 diabetes in a specific neighborhood [28]. Participants at local senior centers [32] and visitors of a social/community center [35] were the study participants in two studies. The other three studies selected participants from various neighborhoods of a city [29,31,33].

Participants in one of the two UK-based studies [29] were required to speak Dutch to meet eligibility criteria, whereas Zandieh et al. [35] included only English-speaking participants. Zhang et al. [34] conducted the study in the UK and China; the participants’ languages were English and Chinese, respectively. Although the study conducted by Chong et al. [28] was based in New South Wales, Australia, it included participants born in English-speaking countries and from Middle Eastern, Asian, and other language-speaking countries. The four studies that were based in China [30,31,33,36] and one study conducted in Spain [32] did not explicitly mention the participants’ language.

### 3.2. Description of UGS Studied

Table 2 also summarizes the type of UGS studied, including “urban green space,” “green space,” “greenspace,” and “parks.” Parks and green spaces were evaluated in three studies [28,32,36]. In the study by Miralles-Guasch et al. [32], parks were specifically defined as green spaces within the city area that were larger than 1 hectare. Neighborhood green spaces, defined as open, undeveloped land with natural vegetation within neighborhoods, were evaluated in four studies [30,31,33,35], further defined by Zhai et al. [30] as parks between 3 and 10 hectares in size. Urban public parks were included in two studies [29,34]. One study examined neighborhood urban parks, defined as publicly owned land within a neighborhood designed to serve the recreation needs of “people living or working within one-half mile radius of the park” [37].

### 3.3. Measures Used in the Studies

#### 3.3.1. PA Measures

Table 3 and Table 4 summarize the association between specific UGS qualities included in the study and the type of PA measured, along with the tools and methods used to collect UGS and PA data. These were categorized into one of the three dimensions of the broader quality domain—availability, accessibility, and attractiveness (Figure 1)—based on the definitions by Biernacka and Kronenberg [22,23].

The nine selected studies measured four PA outcomes: walking, active time in the UGS/park, energy expenditure from PA, and general PA or exercise. Chong et al. [28] measured both walking and moderate-to-vigorous PA (MVPA) in the UGS. Poppe et al. [29] measured both MVPA and light-intensity PA (LPA). Three other studies measured only walking as the outcome [30,31,35]. Active time in the park was measured by two studies [31,32]. One study did not explicitly mention the measured PA [33]. Two studies reported metabolic equivalent (MET) values to measure the impact of PA levels [30,34]. One study examined the recommended level of PA from self-reported national data [36].

Out of nine studies, only one used accelerometers to measure active time in combination with a Global Positioning System (GPS) and Geographic Information System (GIS) [32]. GPS devices record time-stamped movement and location information as geographic coordinates, which can be downloaded and analyzed using GIS software. GPS was also used in the study conducted by Zandieh et al. [35] to measure the average walking activity of the neighborhood participants. Only one study mentioned obtaining informed consent from participants before handing them a GPS to wear on their wrists during their time in the park [32]. Pedometers were used in the study by Zhai et al. [30] to track steps taken by the participants. One study used the System for Observation Play and Recreation in Communities (SOPARC) to enable trained observers to collect PA data in the park [34]. Three studies [28,31,33] collected self-reported PA information.

#### 3.3.2. UGS Measures

Secondary data sources from various periods were used to collate UGS data for analysis. These included data sources with local administrations (e.g., city and council), satellite-based remote sensing images, and other open-source maps (e.g., Baidu map, StreetPro). GIS was used to extract information on proximity, size, shape, land cover type, and other infrastructure. Some quality-related information, such as attractiveness and park safety features, was collected through self-report questionnaires [30,31,34,35].

### 3.4. Relationship Between UGS Quality and PA

Regression analysis was used to evaluate the relationship between UGS quality and PA across all studies (Table 3). All but two studies [31,32] incorporated control variables into their regression analyses to assess the relationship between UGS and PA outcomes.

#### UGS Qualities Studied

Table 4 presents the number of studies that explored UGS qualities within the dimensions of availability, accessibility, and attractiveness domains. The table summarizes the UGS qualities measured within the dimensions of availability (e.g., proximity and size), accessibility (e.g., infrastructure and safety), and attractiveness (e.g., amenities and landscape cover), based on definitions by Biernacka and Kronenberg [22]. Two studies, Zhai et al. [30] and Zhang et al. [34], measured qualities in all three dimensions. Miralles-Guasch et al. [32] evaluated qualities in the availability and attractiveness dimensions. The other studies evaluated qualities only in the availability domain [30,32,34].

**Figure 3:**
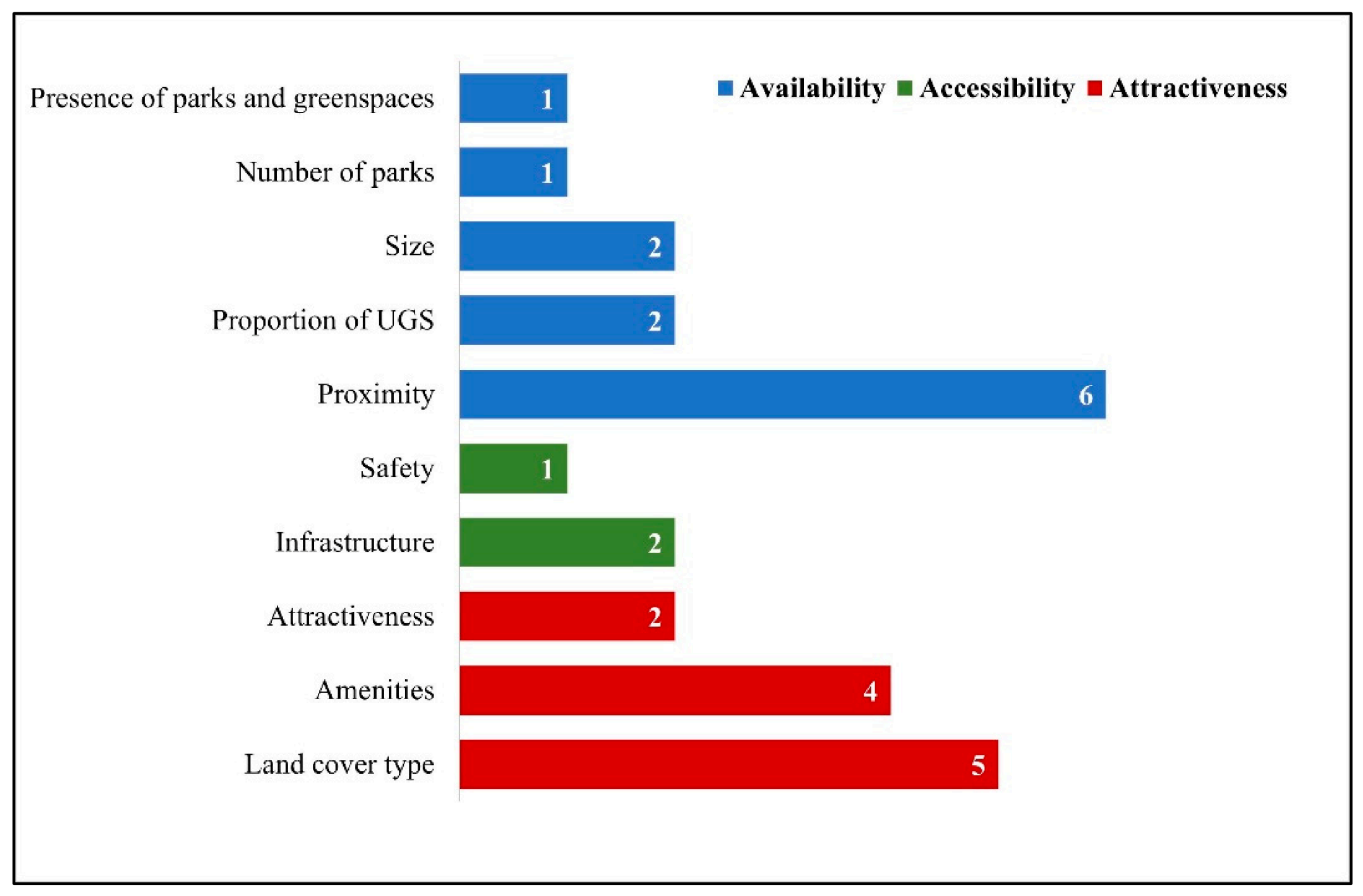
UGS qualities by three dimensions of UGS identified in the included studies.

Figure 3 summarizes the number of UGS qualities explored within the dimensions of availability, accessibility, and attractiveness domains. Overall, only 10 UGS qualities were studied—five within the availability dimension, two within the accessibility domain, and three under attractiveness. Of these, the most frequently explored qualities were: proximity, assessed in six studies [29,31,32,33,34,35]; land cover type, assessed in five different ways across two studies [30,32]; and amenities, assessed four different ways across two studies [30,34].

**Availability:** Availability refers to the extent to which green spaces exist in an area, e.g., the presence and number of UGSs in an area, the distribution of UGS, and the proportion and size of UGS in an area. It also includes the distance from the residential area or proximity to the park. All nine studies included at least one measure of availability. Zandieh et al. [35] evaluated the number of parks within the neighborhood and found no significant relationship between walking and the number of parks. However, the presence and size of UGS were found to be positively related to PA by Huang et al. [36], Zandieh et al. [35], and Zhai et al. [30].

Proximity was evaluated in six studies [29,31,32,33,34,35] with the goal of examining whether the distance to UGS from participants’ residences influences PA patterns among older adults. Three studies [28,29,31] used preset distance buffers to examine the relationship between distance and PA levels among older adults. Chong et al. [28] used 500 m, 1 km, and 2 km buffers around participants’ residences. Liu et al. [31] used buffer zones of 0 to 800 m and more than 800 m to calculate the distance to the nearest neighborhood parks and green spaces. Poppe et al. [29] evaluated the number of parks within 500 m, 1000 m, and 2000 m. No preset distance was used in the other four studies [32,33,34,35].

A mixed-impact result was observed across the six studies. Poppe et al. [29] observed that younger older adults exercised more when more parks were nearby, whereas adults over 75 years exercised less, despite having more parks near their residence. Additionally, no significant relationship between proximity and LPA was found among participants in Poppe et al. [29]. Zhang et al. [34] reported no correlation between distance to a park and PA in Hong Kong, but found a positive correlation between shorter distance to a park and PA patterns in Leipzig. Liu et al. [31] and Miralles-Guasch et al. [32] found a negative correlation between distance to the parks and the time spent walking or being active there. Zandieh et al. [35] did not find any significant relationship between proximity to neighborhood green space and walking. Self-reported PA and distance to the neighborhood park were positively associated in the study by Zhang et al. [33].

**Accessibility:** Accessibility to UGS refers to the ease of accessing the UGS. It includes barrier-free entries, fences, walking paths, bike lanes, car parking spaces, building signage, accessibility adaptations for disabled users, and slopes. Infrastructure such as trail length and park features were evaluated in two studies [30,34]. Zhai et al. [30] found a positive correlation between trail length and walking patterns among older adults. Zhang et al. [34] examined park features in a study conducted across two countries and reported mixed findings on the relationship between park features and active time spent in parks.

The perception of park safety refers to how safe older adults feel in a park environment based on factors such as crime risk, lighting, visibility, and other park users [34]. Zhang et al. [34] examined the relationship between older adults’ perceptions of park safety and their PA patterns and found a positive association in Leipzig but not in Hong Kong. Moreover, none of the associations was significant.

**Attractiveness**: Regarding attractiveness, two studies examined four land cover types and found mixed relationships. Land cover could be natural (i.e., tree cover, bush cover, grass cover) or man-made (i.e., gravel, dirt, paved, or similar). Miralles-Guasch et al. [32] reported mixed associations between PA and land cover type (negative relationship with gravel surfaces, positive relationship with paved surfaces, and no significant association with natural land cover). Zhai et al. [30] found a positive association between older adults’ PA and the number of natural areas in parks, while no significant association was observed between PA and total paved activity area.

The attractiveness domain also included amenities such as the type of activity area, the presence of outdoor fitness equipment, courts, and water bodies. Among these features, activity areas [34] and outdoor fitness equipment [30] were found to positively influence PA among older adults.

Zandieh et al. [35] defined attractiveness as the presence of specific features, such as a café, lake or reservoir, toilet, wildlife, woodland, farm, or conservation area. Zandieh et al. [35] found no significant association between attractiveness and PA. In contrast, Zhang et al. [34] reported mixed findings: no association in Hong Kong but a positive association in Leipzig.

**Figure 4:**
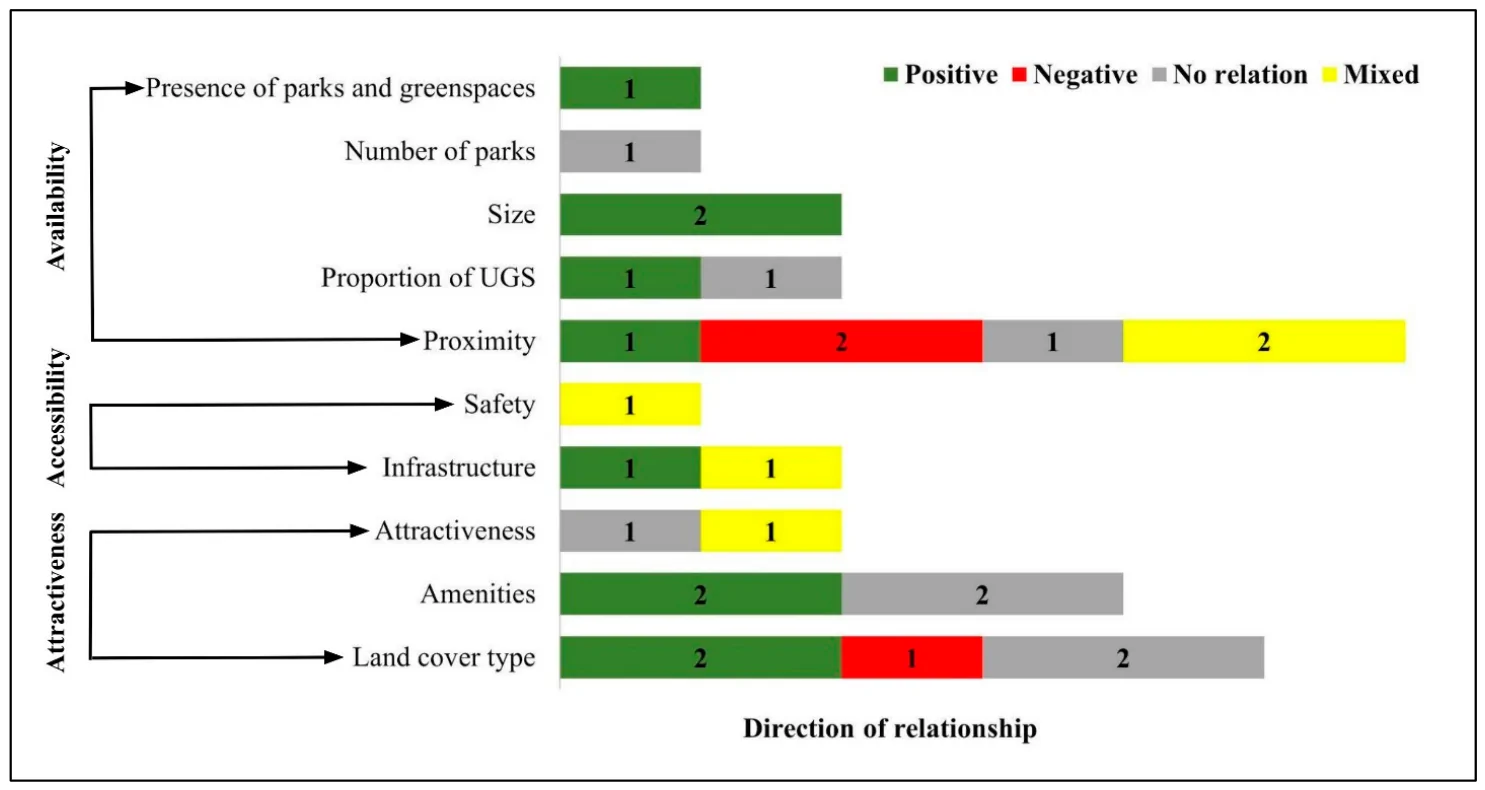
UGS qualities studied and direction of relationships by three dimensions of UGS.

Figure 4 summarizes these findings graphically, showing the relationship between the quality and PA measures as positive (green), negative (red), neutral (gray), or mixed (yellow) within the same study. For example, as noted above, only one of the six studies assessing the proximity measure and PA found a positive association, two found a negative one, one found no association, while two studies had mixed results.

### 3.5. Methodological Rigor

A summary of the assessment of methodological rigor of the eight cross-sectional studies and one longitudinal study are shown in Table 5 and Table 6, respectively. Among the eight cross-sectional studies, two [34,35] met all eight JBI criteria, one study [28] met seven criteria, two [32,36] met six criteria, and the remaining three cross-sectional studies [30,31,33] met five or fewer criteria. The longitudinal study [29] met 10 out of 11 JBI criteria. All studies clearly described the inclusion criteria for the sample population and the study’s subjects and settings, and specified the criteria for measuring exposure validity and reliability. Overall, three studies met 91% or more of the criteria, three met 70–88%, and three met 50–63%.

Although some of the studies scored above 70%, they had important limitations in the control of confounding, outcome assessment, and exposure measurement that may have affected the association between PA and the qualities of UGS. For example, some studies reported using regression models but adjusted only for a limited set of covariates (e.g., age, sex, occupation) while omitting important factors such as socioeconomic status (SES) [34]. The presence of residual confounding and the inherent constraints of cross-sectional designs limit causal interpretation, even when statistical analyses were applied. This is reflected in the relationship between methodological rigor and the strength of the outcome’s relationship (see Supplementary Document S2). For example, across the six studies that examined proximity, two scored less than 70 and the other four scored over 90.

## 4. Discussion

### 4.1. Summary of Findings

This review included nine articles published between 2016 and 2024. Overall, the review found an inconsistent relationship between UGS quality and older adults’ PA. Although some UGS qualities showed positive associations, they were examined too infrequently to support a firm conclusion. In the availability domain, the presence and size of UGS (measured in three studies) were positively associated with older adults’ PA. This aligns with previous research showing that UGS presence and larger green spaces are linked to higher self-reported PA and better health outcomes [20,38]. Proximity to UGS showed inconsistent associations with PA, contradicting the findings of Levy-Storms et al. [20], Kemperman et al. [38], and Chow [39]. However, this may also suggest that proximity alone may not be sufficient to promote PA. Older adults may prefer more attractive UGS over those that are simply closest to them. Even when a park is nearby, poor sidewalk conditions, traffic hazards, and concerns about crime may discourage older adults from using UGS for PA.

In the accessibility domain, findings were mostly self-reported. Infrastructure, such as trail length, showed a positive correlation. However, park features and safety showed inconsistent relationships with PA. Self-reported or perceived safety also showed inconsistent findings in previous studies [40,41,42,43]. Perceived safety can influence older adults’ willingness to use UGS, as perceptions can be shaped by traffic conditions, socioeconomic conditions of the community or neighborhood [43], and other contextual factors like the park location and setting.

The attractiveness domain also demonstrated mixed associations between UGS qualities and older adults’ PA. Land-cover findings varied (negative for gravel, positive for paved, no clear effect for natural cover), while specific amenities such as activity areas, outdoor fitness equipment, and overall attractiveness (visual appeal) showed mixed results. Levy-Storms et al. [20] also reported conflicting relationships between UGS attractiveness and older adults’ PA, while Barnett et al. [41] reported that “aesthetically pleasing scenery and walking-friendly infrastructure” positively correlated with the outdoor PA pattern among older adults.

### 4.2. Heterogeneous Findings

This review identified substantial heterogeneity across studies, which may partly explain the inconsistent associations between UGS quality and PA among older adults. First, the definition of “older adults” varied considerably across studies, with some including participants as young as 45 years. This is important because individuals’ functional capacity and mobility tend to decline with age, which can influence how people use UGS. For example, younger older adults may not have difficulty visiting more distant UGS, whereas adults aged 75 years and above may face mobility limitations that make distance a greater barrier.

Second, the operationalization and measurement of UGS quality varied considerably across studies. Although many studies assessed similar UGS quality dimensions, they differed in how these were defined and measured. Some studies relied on objective measures, such as GIS-based assessment, which was used to measure proximity and size, whereas others used subjective or self-reported measures to capture perceived qualities, including safety and land cover types. Individuals’ perceptions can be influenced by their social and environmental factors, such as SES, and may also vary by age. For example, adults aged 75+ may potentially place greater importance on accessibility, safety, and supportive features than younger older adults [44]. These differences in operationalization and measurement may explain the inconsistent associations observed, particularly for perceived safety and attractiveness.

Third, PA measurement varied across studies, with some investigators using self-reported questionnaires and others employing device-based measures. Self-reported PA is susceptible to recall and social desirability bias, particularly among older adults, potentially leading to overestimation of activity levels and stronger observed associations than those obtained using objective measures [45].

Contextual factors also likely contributed to the heterogeneity across studies, as the included samples were drawn from different countries and socioeconomic settings. These differences may reflect variations in urban design, transportation systems, and cultural norms related to outdoor activity, all of which can shape PA behavior and UGS use. In lower-SES areas, limited access to high-quality UGS and greater perceived or actual safety concerns may shape both objective exposure and subjective perceptions of the UGS environment [46]. As a result, socioeconomic context may have biased subjective reporting of measurements and led to heterogeneous outcomes for the same UGS quality.

### 4.3. Limitations and Recommendations

The strength of this review lies in its focus on quality-related aspects of UGS within the domains of availability, accessibility, and attractiveness. The examination of UGS qualities remains uncommon in studies of older adults’ PA patterns. However, several limitations should be acknowledged. UGS research is multidisciplinary and spans geography, social science, urban planning, and public health. Thus, restricting the search to PubMed, CINAHL, and Web of Science may have excluded relevant studies indexed in Scopus, which was not available at our university. This may explain why no eligible studies from the United States were identified. In addition, limiting inclusion to English-language articles may have reduced the number of eligible studies, particularly given that relatively few papers have examined older adults in relation to UGS quality [47]. Future reviews should broaden database coverage, consider articles written in languages other than English, and include gray literature to improve the comprehensiveness of the evidence base.

None of the studies incorporated transportation-related accessibility features such as bike lanes, parking spaces, directional signage, accessibility accommodations, public transport connections, and road safety measures. Future studies should consider including these features. Evaluating these factors is important for ensuring physical accessibility for older adults who walk and drive to the park [44]. Researchers should also consider social and neighborhood context, including socioeconomic conditions, while evaluating UGS qualities to promote PA among older adults.

Eight of the nine studies in this review used a cross-sectional design, while one used a longitudinal design. Similar patterns have been reported in previous systematic reviews [20,21,41,48]. Although cross-sectional studies are valuable for identifying associations, they are more susceptible to selection, measurement, and confounding biases and do not allow for causal inference.

While quantitative studies can estimate the strength of associations between UGS quality and PA, qualitative methods such as focus groups may provide richer insights into how UGS should be designed. Future researchers should consider a mixed-methods study design that combines quantitative and qualitative approaches to provide a more comprehensive understanding of which UGS qualities are most important in promoting PA among older adults. Community involvement in planning and design would also help ensure that these spaces better reflect local needs and preferences and support older adults’ PA and use of UGS.

## 5. Conclusions

Overall, this review suggests that the association between UGS quality and PA among older adults is context-dependent and sensitive to conceptual and methodological differences. Taken together, these findings indicate that future studies should use more standardized definitions and measurement approaches to improve comparability across settings.

The findings of this systematic review have important implications for age-friendly cities. Planning for older adults should go beyond simply increasing the amount of UGS, adding infrastructure or amenities, or making UGS aesthetically attractive. Instead, planning should be guided by older adults’ perspectives and actual needs to ensure that green spaces are truly usable, encourage regular PA, and support healthy aging.

## Figures and Tables

**Table 1:** Characteristics of the studies included in the review.

Domains	Search Terms
Population	Aged, ageing, aging, elderly, older adults, older person, and senior/s
Intervention	Park, playground, green space, greenspace, sport field, recreation area, public ground, public park, outdoor, greenway, green infrastructure
Comparison	Any
Outcomes	Exercise, physical activity, physical health, walking, moderate-to-vigorous exercise, cycling, biking, bicycling, active play, leisure, sports
Study design	Quantitative studies only

**Table 2:** General characteristics of the included studies.

Study	Location	Study Design	Population	Sample Size	Age Range; Mean Age; % Female	Socioeconomic Status (SES)	Green Space Type
Huang et al., 2018 [36]	Taiwan, China	Cross-sectional	Older adults living in urban areas	2214	≥65 y; 74 y; 54.7%	Level of education: Illiterate 28%; Literate 17%. ≤6 years of education 30.4% ≥7 years of education 24.5%	Parks and green spaces
Chong et al., 2019 [28]	New South Wales, Australia	Cross-sectional, prospective cohort	Residents of the city with T2D	18,094	≥45 y; 59.5 y; 52%	Majority non-English speakers; 1/4 did not complete high school	Parks and green spaces
Miralles-Guasch et al., 2019 [32]	Barcelona, Spain	Cross-sectional	Residents of senior centers	122	Age undefined; 54% aged 65–75 y; 44%	High- and low-income neighborhoods	UGS within the city’s urban continuum, size between 0.5 and 2 ha
Zandieh et al., 2019 [35]	Birmingham, United Kingdom	Cross-sectional	From social centers and eight selected wards	173	≥65; 74.2 y; 57%	High- and low-deprivation areas	Neighborhood green spaces
Zhang et al., 2019 [34]	Hong Kong, China	Cross-sectional	Park users	317	≥60 y; 69.96 y; 42%	Not mentioned	Six randomly selected urban parks in each city
	Leipzig, Germany			311	≥60 y; 72 y; 47.2%		
Zhai et al., 2020 [30]	Shanghai, China	Cross-sectional	Park users	234	≥60 y; 60 y; 43.7%	Not mentioned	15 neighborhood parks, size 3–10 ha
Liu et al., 2020 [31]	Dalian, China	Cross-sectional	From inner city, the fringe of the city and the area between the inner city and the fringe	336	>60 y; equally distributed; 49.7%	Not mentioned	Neighborhood green spaces
Zhang et al., 2021 [33]	Guangzhou, China	Cross-sectional	Residents for more than 6 months	882	>60 y; 79% aged 60–75 y; 56.3%	Not mentioned	Neighborhood parks and squares
Poppe et al., 2022 [29]	Ghent, Belgium	Longitudinal design	431 community-dwelling older adults	Baseline 431; follow-up 147	≥65 y; 72–74 y; 52–54%	Occupational level before retirement, educational level	Urban public parks

**Table 3:** UGS characteristics and PA measures.

	General Categories	Tools Used to Identify or Assess UGS	Statistical Analysis	Factors Adjusted in Statistical Analysis	Type of PA Measure and Tools Used to Measure	UGS Quality Measured	Direction of Effects ^a^	UGS Details
**Availability domain**								
Zandieh et al., 2019 [35]	Number of parks	GIS analysis (proximity, attractiveness, size, and number)	Hierarchical linear regression	SES	Walking; “pedestrian route network” which is the length of all man-made roads and paths, using GIS and GPS devices	Number of parks	(0)	Neighborhood green spaces
Huang et al., 2018 [36]	Presence of parks and green spaces	GIS (buffer distance)	Multilevel hierarchical linear modeling	Median income of the township	Exercise, self-reported	Presence of parks and green spaces	(+)	Parks and green spaces
Chong et al., 2019 [28]	Proportion of UGS	GIS (network analysis)	Multiple regression	Sociodemographic characteristics (age, gender, country of birth) and area-level deprivation score	Walking, moderate-to-vigorous PA (MVPA); self-reported	% of GS within all buffer polygon-based road network buffers	(0)	Parks and green spaces
Miralles-Guasch et al., 2019 [32]	Proportion of UGS	GIS	Mixed-effects multilevel regression	Not clear	Active time; GPS devices, accelerometers, and PALMS software	Proportion of UGS	(+)	Parks and gardens within the city’s urban continuum < 1 ha
Poppe et al., 2022 [29]	Proximity	GIS (buffer distance)	Generalized Linear Mixed Models (GLMMs), longitudinal analysis, logistic regression	Age, occupational class, physical functioning	Moderate-to-vigorous physical activity (MVPA), light-intensity physical activity (LPA)	Number of parks within 500 m, 1000 m, and 2000 m	MVPA: (+) < 75 yrs (younger older adults), (−) for >75 yrs; LPA: (0)	Urban public parks
Liu et al., 2020 [31]	Proximity	GIS (network analysis)	Mixed multinomial logit model	Not clearly mentioned	Walking and self-reported active time	Proximity (0–800) m	(−)	Neighborhood green spaces
Miralles-Guasch et al., 2019 [32]	Proximity	GIS	Mixed-effects multilevel regression	Not clear	Active time; GPS devices, accelerometers, and PALMS software	Distance	(−)	Parks and gardens within the city’s urban continuum < 1 ha
Zandieh et al., 2019 [35]	Proximity	GIS analysis (proximity, attractiveness, size, and number)	Hierarchical (also known as multilevel) linear regression	SES	Walking: “pedestrian route network” which is the length of all man-made roads and paths, using GIS and GPS devices	Proximity	(0)	Neighborhood green spaces
Zhang et al., 2019 [34]	Proximity	Self-reported questionnaire	Hierarchical regression	Hong Kong: age, gender; Leipzig: marital status	Active time; SOPARC	Proximity	Hong Kong: (0); Leipzig: (+)	
Zhang et al., 2021 [33]	Proximity	GIS (buffer distance)	Linear regression	Age, gender, marital status, education level, income, lifestyle, and individual preferences including travel model, smoking, and drinking	Self-reported PA; MOS 36-Item Short-Form Health Survey (SF-36)	Distance	(+)	Neighborhood parks and squares
Zandieh et al., 2019 [35]	Size	GIS analysis (proximity, attractiveness, size, and number)	Hierarchical linear regression	SES	Walking; “pedestrian route network” which is the length of all man-made roads and paths, using GIS and GPS devices	Size	(+)	Neighborhood green spaces
Zhai et al., 2020 [30]	Size		Multiple stepwise regression analyses	Demographic attributes	Pedometer; self-reported energy expenditure	Park area	(+)	15 neighborhood parks between 3 and 10 ha
**Accessibility domain**								
Zhai et al., 2020 [30]	Infrastructure	Self-reported	Multiple stepwise regression analyses	Demographic attributes	Pedometer; self-reported energy expenditure	Trail length	(+)	15 neighborhood parks between 3 and 10 ha
Zhang et al., 2019 [34]	Infrastructure	Self-reported questionnaire	Hierarchical regression	Hong Kong: age, gender; Leipzig: marital status	Active time; SOPARC	Park features	Hong Kong: (0); Leipzig: (+)	
Zhang et al., 2019 [34]	Safety	Self-reported questionnaire	Hierarchical regression	Hong Kong: age, gender; Leipzig: marital status	Active time; SOPARC	Park safety	Hong Kong: (0); Leipzig: (+)	6 randomly selected urban parks in each city
**Attractiveness domain**								
Zhang et al., 2019 [34]	Amenities	Self-reported questionnaire	Hierarchical regression	Hong Kong: age, gender; Leipzig: marital status	Active time; SOPARC	Types of activity area	Hong Kong: (+); Leipzig: (+)	6 randomly selected urban parks in each city
Miralles-Guasch et al., 2019 [32]	Land cover type	GIS	Mixed-effects multilevel regression	Not clear	Active time; GPS devices, accelerometers, and PALMS software	Forest, shrubland, grassland,	(0)	Parks and gardens within the city’s urban continuum < 1 ha
Zhai et al., 2020 [30]	Land cover type	Self-reported	Multiple stepwise regression analyses	Demographic attributes	Pedometer; self-reported energy expenditure	Total paved activity zone	(0)	15 neighborhood parks between 3 and 10 ha
Zhai et al., 2020 [30]	Amenities	Self-reported	Multiple stepwise regression analyses	Demographic attributes	Pedometer; self-reported energy expenditure	Presence of outdoor fitness equipment	(+)	15 neighborhood parks between 3 and 10 ha
Zhai et al., 2020 [30]	Amenities	Self-reported	Multiple stepwise regression analyses	Demographic attributes	Pedometer; self-reported energy expenditure	Presence of court	(0)	15 neighborhood parks between 3 and 10 ha
Zandieh et al., 2019 [35]	Attractiveness	GIS analysis (proximity, attractiveness, size, and number)	Hierarchical linear regression	SES	Walking; “pedestrian route network” which is the length of all man-made roads and paths, using GIS and GPS devices	Attractiveness	(0)	Neighborhood green spaces
Zhang et al., 2019 [34]	Attractiveness	Self-reported questionnaire	Hierarchical regression	Hong Kong: age, gender; Leipzig: marital status	Active time; SOPARC	Attractiveness (visual appeal and overall pleasantness)	Hong Kong: (0); Leipzig: (+)	6 randomly selected urban parks in each city
Zhai et al., 2020 [30]	Amenities	Self-reported	Multiple stepwise regression analyses	Demographic attributes	Pedometer; self-reported energy expenditure	Presence of water body/feature	(0)	15 neighborhood parks between 3 and 10 ha
Miralles-Guasch et al., 2019 [32]	Land cover type	GIS	Mixed-effects multilevel regression	Not clear	Active time; GPS device, accelerometers, and PALMS software	Pavement	(+)	Parks and gardens within the city’s urban continuum < 1 ha
Miralles-Guasch et al., 2019 [32]	Land cover type	GIS	Mixed-effects multilevel regression	Not clear	Active time; GPS device, accelerometers, and PALMS software	Gravel	(−)	Parks and gardens within the city’s urban continuum < 1 ha
Zhai et al., 2020 [30]	Land cover type	Self-reported	Multiple stepwise regression analyses	Demographic attributes	Pedometer; self-reported energy expenditure	Total natural area in the park	(+)	15 neighborhood parks between 3 and 10 ha

^a^ Direction of effect is presented as + (positive association), − (negative association), 0 (no association).

**Table 4:** Accessibility domains and PA measures by included studies.

Study	UGS			PA			
	Availability	Accessibility	Attractiveness	PA or Exercise	Walking	Active Time	Energy Expenditure
Huang et al., 2018 [36]	GIS (buffer distance)			Self-reported			
Chong et al., 2019 [28]	GIS (network analysis)				Self-reported		
Miralles-Guasch et al., 2019 [32]	GIS (generate UGS location)		GIS (generate UGS location)			GPS device, accelerometer, and PALMS software	
Zandieh et al., 2019 [35]	GIS (network analysis)		GIS (network analysis)		GIS		
Zhang et al., 2019 [34]	Self-reported			System for Observation Play and Recreation in Communities (SOPARC)			
Zhai et al., 2020 [30]	Self-reported						Pedometer; self-reported
Liu et al., 2020 [31]	GIS (network analysis)					Self-reported	
Zhang et al., 2021 [33]	GIS (buffer distance)			Self-reported			
Poppe et al., 2022 [29]	GIS (buffer distance)			Accelerometer			
Total	9	2	4	3	2	3	1

**Table 5:** Study quality assessment for cross-sectional studies.

Study	Were the Criteria for Inclusion in the Sample Clearly Defined?	Were the Study Subjects and the Setting Described in Detail?	Was the Exposure Measured in a Valid and Reliable Way?	Were Objective, Standard Criteria Used for Measurement of the Condition?	Were Confounding Factors Identified?	Were Strategies to Deal with Confounding Factors Stated?	Were the Outcomes Measured in a Valid and Reliable Way?	Was Appropriate Statistical Analysis Used?	Total Criteria Met	% of Criteria Met
Huang et al., 2018 [36]	1	1	0	1	1	1	0	1	6	75
Chong et al., 2019 [28]	1	1	1	1	1	1	0	1	7	87.5
Miralles-Guasch et al., 2019 [32]	1	1	1	1	0	0	1	1	6	75
Zandieh et al., 2019 [35]	1	1	1	1	1	1	1	1	8	100
Zhang et al., 2019 [34]	1	1	1	1	1	1	1	1	8	100
Zhai et al., 2020 [30]	1	1	1	1	1	1	0	-	5	62.5
Liu et al., 2021 [31]	1	1	1	0	0	0	0	1	4	50
Zhang et al., 2021 [33]	1	1	1	0	1	1	0	-	5	62.5

**Table 6:** Study quality assessment for the longitudinal study.

Study	Were the Two Groups Similar and Recruited from the Same Population?	Were the Exposures Measured Similarly to Assign People to Both Exposed and Unexposed Groups?	Was the Exposure Measured in a Valid and Reliable Way?	Were Confounding Factors Identified?	Were Strategies to Deal with Confounding Factors Stated?	Were the Groups/Participants Free of the Outcome at the Start of the Study (or at the Moment of Exposure)?	Were the Outcomes Measured in a Valid and Reliable Way?	Was the Follow-Up Time Reported and Sufficient to Be Long Enough for Outcomes to Occur?	Was the Follow-Up Complete, and If Not, Were the Reasons for the Loss to Follow-Up Described and Explored?	Were Strategies to Address Incomplete Follow-Up Utilized?	Was Appropriate Statistical Analysis Used?	Total Criteria Met	% of Criteria Met
Poppe et al., 2022 [29]	1	1	1	1	1	1	1	1	0	1	1	10	91

## Data Availability

Articles and extraction tables are available from N.S.

## Supplementary Materials

The following supporting information can be downloaded at: https://www.mdpi.com/article/10.3390/ijerph23080970/s1, S1: Search strategies of all databases; S2: Methodological quality and direction of relationship. S3: PRISMA 2020 abstract checklist 2026 [26]. S4: PRISMA 2020 checklist Quality 2026 [26].
