# Supplementary material for: Quality of Urban Green Space for Older Adults to Promote Physical Activity: A Systematic Review
Source: Int J Environ Res Public Health. 2026 Jul 27;23(8):970. doi: 10.3390/ijerph23080970 (PMC13512371; doi:10.3390/ijerph23080970)
Supplement: Supplementary file 1 [file ijerph-23-00970-s001.zip › S1-Search strategies of all databases-new.pdf]

Quality of urban green spaces for older adults to promote physical activity: A systematic review

Search Strings

**PubMed**	**Search Strings**
S1	("Aged"[Mesh]) OR elderly[Title/Abstract] OR "older adults"[Title/Abstract] OR Senior[Title/Abstract] OR "older population"[Title/Abstract] OR (Aging[Title/Abstract])
S2	(("Parks, Recreational"[Mesh]) OR Park[Title/Abstract] OR parks[Title/Abstract] OR "play ground"[Title/Abstract] OR playground[Title/Abstract] OR "sport field"[Title/Abstract] OR "recreation area"[Title/Abstract] OR "public ground"[Title/Abstract] OR "public parks" [Title/Abstract] OR "public park"[Title/Abstract] OR outdoor[Title/Abstract] OR "built environment"[Title/Abstract]) OR ((((greenspace[Title/Abstract]) OR (green space[Title/Abstract])) OR (green infrastructure[Title/Abstract])) OR (greenway[Title/Abstract]))
S3	("Exercise"[Mesh]) OR (Walking[Mesh]) OR Physical activity[Title/Abstract] OR exercise[Title/Abstract] OR moderate-to-vigorous[Title/Abstract] OR MVPA[Title/Abstract] OR physical health[Title/Abstract] OR sedentary[Title/Abstract] OR METs[Title/Abstract] OR metabolic equivalent task[Title/Abstract] OR walking[Title/Abstract]
S4	S1 AND S2 AND S3)

**CINAHL**	**Search Strings**
S1	TI (elderly or older adults or aging or senior or seniors or older people or aged 65 or 65+) OR AB (older adults or aging or senior or seniors or older people or aged 65 or 65+)
S2	TI (park or parks or public park or urban park or green infrastructure or greenway or greenspace or green space or recreational area or playground or built environment or urban green space or urban nature or urban forest) OR AB (park or parks or “public park” or “urban park” or “green infrastructure” or greenway or greenspace or “green space” or “recreational area” or playground or “built environment” or “urban green space” or “urban nature” or “urban forest”)
S3	TI(walking or exercise or physical activity or physical exercise or exercising or moderate-to-vigorous" or MVPA or metabolic equivalent task) OR AB (walking or exercise or physical activity or physical exercise or exercising or moderate-to-vigorous" or MVPA or metabolic equivalent task)
S4	S1 AND S2 AND S3

Quality of urban green spaces for older adults to promote physical activity: A systematic review

Web of Science	Search Strings
1	TI=("elderly" OR "aged" OR "older" OR "elder" OR "elderly people" OR "old people" OR "senior") OR AB= ("elderly" OR "aged" OR "older" OR "elder" OR "elderly people" OR "old people" OR "senior")
2	TI=("Parks" OR "Park" OR "play ground" OR "playground" OR "sport field" OR "recreation area" OR "public ground" OR "outdoor" OR "built environment" OR "greenspace" OR "green space" OR "green infrastructure" OR "greenway") OR AB=(("Parks" OR "Park" OR "play ground" OR "playground" OR "sport field" OR "recreation area" OR "public ground" OR "outdoor" OR "built environment" OR "greenspace" OR "green space" OR "green infrastructure" OR "greenway"))
3	TI=("Physical activity" OR "exercise" OR "moderate-to-vigorous" OR "MVPA" OR "physical health" OR "sedentary" OR "metabolic equivalent task" OR "walking") OR AB=("Physical activity" OR "exercise" OR "moderate-to-vigorous" OR "MVPA" OR "physical health" OR "METs" OR "metabolic equivalent task" OR "walking")
4	#1 AND #2 AND #3
