# Supplementary material for: Quality of Urban Green Space for Older Adults to Promote Physical Activity: A Systematic Review
Source: Int J Environ Res Public Health. 2026 Jul 27;23(8):970. doi: 10.3390/ijerph23080970 (PMC13512371; doi:10.3390/ijerph23080970)
Supplement: Supplementary file 1 [file ijerph-23-00970-s001.zip › S2-Methodological quality and direction of relationship.pdf]

S2a. Qualities Examined in Availability Dimension with Study Quality

**General categories**	**Direction of relationship**				
	**Number of qualities**	**Positive (% JBI items met)**	**Negative (% JBI items met)**	**No relation (% JBI items met)**	**Mixed (% JBI items met)**
Presence of parks and greenspaces	1	75			
Number of parks	1			100	
Size	2	100 and 62.25			
Proportion of UGS	2	75		87.5	
Proximity	6	62.5	50 and 75	100	91 and 100

S2b. Qualities Examined in Accessibility Dimension with Study

General categories	Direction of relationship				
	Number of qualities	Positive (% JBI items met)	Negative (% JBI items met)	No relation (% JBI items met)	Mixed (% JBI items met)
Safety	1				100
Infrastructure	2	62.5			100

S2c. Qualities Examined in Attractiveness Dimension

General categories	Direction of relationship				
	Number of qualities	Positive (% JBI items met)	Negative (% JBI items met)	No relation (% JBI items met)	Mixed (% JBI items met)
Attractiveness	2			100	100
Amenities	4	100 and 62.5		62.5 and 62.5	
Land cover type	5	75 and 62.5	75	75 and 62.5	
